# Selective Engagement of FcγRIV by a M2e-Specific Single Domain Antibody Construct Protects Against Influenza A Virus Infection

**DOI:** 10.3389/fimmu.2019.02920

**Published:** 2019-12-12

**Authors:** Dorien De Vlieger, Katja Hoffmann, Inge Van Molle, Wim Nerinckx, Lien Van Hoecke, Marlies Ballegeer, Sarah Creytens, Han Remaut, Hartmut Hengel, Bert Schepens, Xavier Saelens

**Affiliations:** ^1^VIB-UGent Center for Medical Biotechnology, VIB, Ghent, Belgium; ^2^Department of Biomedical Molecular Biology, Ghent University, Ghent, Belgium; ^3^Department of Biochemistry and Microbiology, Ghent University, Ghent, Belgium; ^4^Institute of Virology, Medical Center, Faculty of Medicine, University of Freiburg, Freiburg, Germany; ^5^Structural Biology Brussels, Vrije Universiteit Brussel, Brussels, Belgium; ^6^VIB-VUB Center for Structural Biology, Brussels, Belgium

**Keywords:** influenza, matrix protein 2 ectodomain, single domain antibody, Fcγ receptor, effector functions

## Abstract

Lower respiratory tract infections, such as infections caused by influenza A viruses, are a constant threat for public health. Antivirals are indispensable to control disease caused by epidemic as well as pandemic influenza A. We developed a novel anti-influenza A virus approach based on an engineered single-domain antibody (VHH) construct that can selectively recruit innate immune cells to the sites of virus replication. This protective construct comprises two VHHs. One VHH binds with nanomolar affinity to the conserved influenza A matrix protein 2 (M2) ectodomain (M2e). Co-crystal structure analysis revealed that the complementarity determining regions 2 and 3 of this VHH embrace M2e. The second selected VHH specifically binds to the mouse Fcγ Receptor IV (FcγRIV) and was genetically fused to the M2e-specific VHH, which resulted in a bi-specific VHH-based construct that could be efficiently expressed in *Pichia pastoris*. In the presence of M2 expressing or influenza A virus-infected target cells, this single domain antibody construct selectively activated the mouse FcγRIV. Moreover, intranasal delivery of this bispecific FcγRIV-engaging VHH construct protected wild type but not *Fc*γ*RIV*^−/−^ mice against challenge with an H3N2 influenza virus. These results provide proof of concept that VHHs directed against a surface exposed viral antigen can be readily armed with effector functions that trigger protective antiviral activity beyond direct virus neutralization.

## Introduction

Influenza A virus infections are a major recurrent cause of seasonal respiratory tract infections. The best way to prevent influenza disease is considered to be vaccination. However due to the accumulation of point mutations in the viral hemagglutinin (HA) and neuraminidase (NA) genes, human influenza vaccines need to be reformulated and administered regularly based on the prediction of the circulating strains (1). The variable effectiveness and the long manufacturing timeline encourage the development of more broadly protective vaccines. Antivirals, such as oseltamivir and baloxavir marboxil, have been licensed for the prophylaxis and treatment of uncomplicated influenza, but the risk of selecting drug resistant viruses limits their widespread use (2, 3).

The 23 amino acid residues long M2 ectodomain (M2e) is highly conserved among the different influenza A virus subtypes and thus represents an attractive target for broadly protective prophylactic vaccine strategies as well as for antibody-based antiviral biologicals [reviewed in Saelens (4)] (4, 5). Various studies using different vaccine formats have demonstrated that M2e-based vaccination can provide broad protection in animal models of influenza A and that this protection is antibody mediated (6–9). Next to vaccines, a therapeutic intervention with an intravenously administered recombinant human IgG1 monoclonal antibody directed against the M2 N-terminus was found to reduce the symptoms in human volunteers that had been infected with an H3N2 virus (10). Furthermore, this phase 2a trial showed that the antibody treatment was associated with a trend toward reduced viral shedding from the nasal mucosa, and no anti-M2e escape mutants could be detected. Finally, intravenous administration of engineered, so called bi-specific T cell engagers that comprise a M2e-specific single chain variable fragment that is linked to a CD3ε-specific single chain variable fragment, could protect mice against an otherwise lethal influenza A virus challenge (11).

Fc gamma receptor (FcγR) interactions are essential for the protective activity of M2e-specific antibodies (12–14). FcγRs are type I membrane proteins that are expressed on different innate immune cells, including macrophages, neutrophils, natural killer cells and dendritic cells (15, 16). In mice, FcγRs are characterized by the presence of an immunoreceptor tyrosine-based activation motif (ITAM) in the cytoplasmic portion of the common γ chain that is associated with the activating FcγRI, FcγRIII and FcγRIV, or by an immunoreceptor tyrosine-based inhibition motif (ITIM) in the cytoplasmic portion of the inhibitory FcγRIIb (17–19). These receptors differ in their affinities for the different IgG isotypes (16, 20). Previously, we have shown that protection by M2e-specific mouse IgG1 requires FcγRIII while IgG2a isotypes can protect by any of the three activating FcγRs (13).

Since the reported discovery of heavy chain-only antibodies in camelids in 1993, recombinant single domain proteins comprising the variable domain of these antibodies (VHHs also known as Nanobodies®) have been used in numerous therapeutic applications (21). In the context of viral infections, various virus-neutralizing VHHs have been described that can interfere with different steps in the viral life cycle (22). Due to their outstanding stability and solubility, as well as their small size (~15 kDa), ease of production and formatting flexibility, they are highly versatile building blocks for the development of new antivirals. Next to a direct antiviral effect, VHHs can also easily be formatted (e.g., by generating Fc fusions) to recruit host effector functions (23). These features, combined with the possibility to deliver therapeutic VHHs into the lung environment, and maintained stability after prolonged storage, make VHH-based anti-influenza biologicals especially attractive for epidemic as well as pandemic preparedness plans (24–27).

In this study we explored a new strategy to engage host cell effector functions to combat influenza A. This strategy is based on a tail-to-head genetic fusion of two VHHs, one that selectively binds to FcγRIV and a second one that is specific for M2e. The resulting bi-specific construct can be efficiently expressed in *Pichia pastoris* cells and protects mice against an otherwise lethal influenza A virus infection by simple intranasal delivery.

## Materials and Methods

### Cell Lines and Culture Conditions

HEK293T cells (a gift from Dr M. Hall, University of Birmingham, Birmingham, UK) and HEK293T cells stably transfected with influenza M2 (28) were cultured in Dulbecco's modified Eagle's medium supplemented with 10% of fetal calf serum, 2 mM of l-glutamine, 0.4 mM of Na-pyruvate, non-essential amino acids, 100 U/ml of penicillin and 10 μM amantadine for the M2 expressing HEK cells. Madin-Darby canine kidney (MDCK) cells were cultured in Dulbecco's modified Eagle's medium supplemented with 10% of fetal calf serum, 2 mM of l-glutamine, non-essential amino acids and 100 U/ml of penicillin. Mf4/4 cells (an immortalized cell line of spleen macrophages derived from C57BL/6 mice) were grown in RPMI 1640 medium, supplemented with 10% of fetal calf serum, 2 mM of l-glutamine, 0.4 mM of Na-pyruvate, non-essential amino acids, 50 mM 2-mercaptoethanol, 25 mM Hepes and 100 U/ml of penicillin (29). Cloning of Fc**γ**R-ζ constructs, the generation of Fc**γ**R-ζ BW5147 reporter cells and the culture conditions were similar as reported previously (30, 31).

### Production of Recombinant Mouse FcγRIV Protein

Recombinant FcγRIV protein was produced by transient transfection of subconfluently grown FreesStyle™293-F cells (ThermoFisher scientific) with pCAGGs expression vectors encoding the ectodomain of FcγRIV (amino acids 1-201) coupled to a C-terminal 6XHis tag. Recombinant FcγRIV protein was purified from the supernatant 6 days after transfection, using a 1 ml HisTrap HP column (GE Healthcare). Fractions containing FcγRIV protein were pooled and concentrated with a Vivaspin column (5 kDa cutoff, GE Healthcare) and then further purified by gel filtration on a Superdex 75 column. Fractions containing FcγRIV protein were pooled and concentrated. Purity was evaluated by SDS-PAGE followed by Coomassie blue staining.

### Isolation of M2e-Binding, VHH-Displaying Phages

A llama was immunized 6 times at weekly intervals subcutaneously with 150 μg M2e-tGCN4 (28) in the presence of Gerbu LQ#3000 adjuvant. Immunizations and handling of the llama were performed according to directive 2010/63/EU of the European parliament for the protection of animals used for scientific purposes and approved by the Ethical Committee for Animal Experiments of the Vrije Universiteit Brussel (permit No. 13-601-1). Five days after the last immunization, blood was collected and lymphocytes were prepared. Total RNA was extracted and used as template for the first strand cDNA synthesis with oligodT primer. The VHH encoding sequences were amplified from the cDNA and cloned into the *Pst*I and *Not*I sites of the phagemid vector pMECS. In this vector, the VHH coding sequence is followed by a linker, an HA- and 6xHis tag (AAAYPYDVPDYGSHHHHHH). Electro-competent *E.coli* TG1 cells were transformed with the recombinant pMECS vector resulting in a VHH library of about 10^8^ independent transformants. A library of VHH-presenting phages was obtained after infection with VCS M13 helper phages. Two different panning strategies were used. In the first strategy, phages were added to 20 μg of immobilized M2e-tGCN4 in panning round 1 and 20 μg of human H3N2 peptide (SLLTEVETPIRNEWGCRCNDSSD) in panning round 2. In the second strategy, phages were first added to 25 × 10^6^ HEK293T cells to deplete potential binders to determinants on these cells. The unbound phages were next added to 25 × 10^6^ HEK293T cells stably transfected with influenza M2, to enrich for M2-specific phages. To avoid internalization of the target antigen, all steps were performed at 4°C. After washing, retained phages were eluted by pH elution with TEA-solution (14% triethylamine (Sigma) pH 10) for 10 min. A solution of 1 M Tris-HCl pH 8 was used to lower the pH of the eluted phage solution. The enrichment relative to panning on the negative control antigen, was determined by infecting TG1 cells with 10-fold serial dilutions of the phages after which the bacteria were plated on LB agar plates with 100 μg/ml ampicillin and 1% glucose.

### Isolation of FcγRIV-Binding, VHH-Displaying Phages

The FcγRIV specific VHHs were isolated form a library that was part of a study described elsewhere by Deschacht et al. (32). In brief, a llama was immunized six times at weekly intervals with 10^8^ immature murine bone marrow-derived dendritic cells. A library of VHH-presenting phages was obtained as described above. FcγRIV specific VHHs were enriched after three panning rounds on 20 μg of immobilized FcγRIV protein.

### Periplasmic ELISA Screen to Identify M2e- and FcγRIV-Specific VHHs

After panning, individual pMEC colonies were randomly selected for further analysis by ELISA for the presence of M2e- and FcγRIV-specific VHHs in their periplasm. To prepare periplasmic extract, individual colonies were inoculated in 2 ml of terrific broth (TB) medium with 100 μg/ml ampicillin in 24-well deep well plates. After 5 h incubation isopropyl β-d-1-thiogalactopyranoside (IPTG) (1 mM) was added to induce VHH expression. After overnight incubation at 37°C, bacterial cells were pelleted and resuspended in 200 μl TES buffer (0.2 M Tris-HCl pH 8, 0.5 mM EDTA, 0.5 M sucrose) and incubated at 4°C for 30 min. An osmotic shock was induced by adding 300 μl of water. After 1 h incubation at 4°C followed by centrifugation, the periplasmic extract was collected. VHH-containing periplasmic extracts were then tested for binding to either M2e-tGCN4 (28), human H3N2 M2e peptide (SLLTEVETPIRNEWGCRCNDSSD) or recombinant mouse FcγRIV protein. Briefly, wells of microtiter plates were coated overnight with either 100 ng M2e-tGCN4, 100 ng mouse FcγRIV protein, bovine serum albumin (BSA, Sigma-Aldrich) at 4°C or 100 ng human H3N2 M2e peptide at 37°C. The coated plates were blocked with 5% milk powder in phosphate buffered saline (PBS) and 100 μl of the periplasmic extract was added to the wells. Bound VHHs were detected with anti-HA mAb (1/2000, MMS-101P Biolegend) followed by horseradish peroxidase (HRP)-linked anti-mouse IgG (1/2000, NXA931, GE Healthcare). All periplasmic fractions, which resulted in OD_450_ values of the antigen coated wells that were at least two times higher than the OD_450_ values obtained in BSA coated wells, were selected. DNA of the selected colonies was isolated using the QIAprep Spin Miniprep kit (Qiagen) and sequenced using the primer MP057(5′-TTATGCTTCCGGCTCGTATG-3′).

### VHH Expression in *Pichia pastoris*

The VHH encoding sequence was amplified by PCR using the following forward and reverse primer (5′-GGC GGG TAT CTC TCG AGA AAA GGC AGG TGC AGC TGC AGG AGT CTG GG-3′) and (5′- CTA ACT AGT CTA GTG ATG GTG ATG GTG GTG GCT GGA GAC GGT GAC CT GG-3′). The PCR fragments were then cloned between the *Xho*I and *Spe*I sites in the pKai61 expression vector [described by Schoonooghe et al. (33)]. In the vector, the VHHs sequences containing a C-terminal 6XHis tag sequence are under control of the methanol inducible AOX1 promotor and in frame with a modified version of the *S. cerevisiae* α-mating factor prepro signal sequence. The vector contains a Zeocine resistance marker for selection in bacteria as well as in yeast cells. The vectors were linearized by *Pme*I and transformed in the *P. pastoris* strain GS115 using the condensed transformation protocol described by Lin-Cereghino et al. (34) After transformation, the yeast cells were plated on YPD plates (1% (w/v) yeast extract, 2% (w/v) peptone, 2% (w/v) dextrose, and 2% (w/v) agar) supplemented with zeocin (100 μg/ml) for selection.

### VHH Production and Purification

The transformed *P. pastoris* clones were first analyzed for VHH expression in 2 ml cultures. On day one, 2–5 clones of each construct were inoculated in 2 ml of YPNG medium (2% pepton, 1% Bacto yeast extract, 1.34% YNB, 0.1 M potassium phosphate pH 6, 1% glycerol) with 100 μg/ml Zeocin (Life Technologies) and incubated at 28°C for 24 h. The next day, the cells were pelleted by centrifugation and the medium was replaced by YPNM medium (2% pepton, 1% Bacto yeast extract, 1.34% YNB, 0.1 M potassium phosphate pH 6.0, 1% methanol). Cultures were incubated at 28°C and 50 μl of 50% methanol was added at 16, 24, and 40 h. After 48 h, the supernatant was collected and the presence of soluble VHHs in the supernatant was verified using SDS-PAGE and subsequent Coomassie Blue staining. Production was scaled up (300 ml) for the transformants with the highest levels of VHH in the medium. Growth and methanol induction conditions and harvesting of medium were similar as mentioned above for the 2 ml cultures. The secreted VHHs in the medium were precipitated by ammonium sulfate (NH_4_)_2_SO_4_ precipitation (80% saturation) for 4 h at 4°C. The insoluble fraction was pelleted by centrifugation at 20,000 g and resuspended in 10 ml binding buffer (20 mM NaH_2_PO_4_ pH 7.5, 0.5M NaCl and 20 mM imidazole pH 7.4). The VHHs were purified from the solution using a 1 ml HisTrap HP column (GE Healthcare). Bound VHHs were eluted with a linear imidazole gradient starting from 20 mM and ending at 500 mM imidazole in binding buffer over a total volume of 20 ml. VHH containing fractions were pooled and concentrated with a Vivaspin column (5 kDa cutoff, GE Healthcare) and then further purified by gel filtration (Superdex 75) in PBS buffer. Fractions containing VHH were again pooled and concentrated. Purity was evaluated by SDS-PAGE followed by Coomassie blue staining.

### Enzyme-Linked Immunosorbent Assay

Wells of microtiter plates were coated overnight with either 100 ng M2e-tGCN4, 100 ng BM2e-tGCN4, 100 ng M2e peptide or 100 ng mouse FcγRIV protein. The coated plates were blocked with 5% milk powder in phosphate buffered saline (PBS) and dilution series of the VHHs were added to the wells. In the M2e ELISA, bound VHHs were detected with mouse anti-Histidine Tag antibody (MCA1396, Abd Serotec) followed by horseradish peroxidase (HRP)-linked anti-mouse IgG (1/2000, NXA931, GE Healthcare). In the ELISA with coated recombinant FcγRIV protein, binding was detected with a HRP conjugated rabbit anti-camelid VHH antibody (A01861-200, GenScript). After washing 50 μl of TMB substrate (Tetramethylbenzidine, BD OptETA) was added to every well. The reaction was stopped by addition of 50 μl of 1M H_2_SO_4_, after which the absorbance at 450 nM was measured with an iMark Microplate Absorbance Reader (Bio Rad).

### Crystallization of M2e-VHH-23m in Complex With M2e Peptide

For crystallization, the purified M2e-VHH-23m was concentrated to 20 mg/ml. The M2e peptide was added in a 1.2 times excess. Crystallization screens were set up in sitting drop vapor diffusion at 20°C. Crystals grown in the Jenna Classic screen, in 30% ethanol, 10% PEG6000, 100 mM Na acetate were cryoprotected using fluorosilicone and flash frozen in liquid nitrogen. X-ray data were collected at the i03 beamline of the Diamond Light Source synchrotron facility and processed using XDS.^47^

The structure of the M2e-VHH-23m-M2e peptide complex was solved using the structure of another nanobody (PDB code 5HGG) as search model for molecular replacement, using the Phaser program from the CCP4 crystallographic software suite (35, 36). The M2e-VHH-23m model was built automatically using Autobuild from the Phenix crystallographic software suite (37). The initial model was further built manually in Coot and refined using phenix.refine and Refmac (38–40). Data collection parameters, as well as processing and refinement statistics are shown in Table 1. The crystal structure has been deposited in the Protein Data Bank (PDB) and is available with accession code 6S0Y.

**Table 1:** Data collection statistics and refinement parameters.

**Data collection**	
Synchrotron	Diamond Light Source
Beamline	i03
Wavelength, Å	0.97965
**Data processing**	
Space group	P 21 21 2
Cell parameters, Å (α = β = γ = 90°)	57.47 94.76 53.06
Resolution, Å (outer shell) ^(c)^	53.06–1.80 (1.83–1.80)
Total reflections	35827 (17918)
No. of unique reflections	27557 (1359)
Completeness	99.97 (100)
Multiplicity	13.0 (13.2)
*R*_pim_, %	4.4 (66.6)
CC_1/2_, %	99.8 (57.1)
<*I*/σ(*I*)>	11.0 (1.1)
Mosaicity, °	0.063°
**Refinement**	
Resolution range, Å	47.38–1.81
No. of reflections	25859
Percentage observed	99.96
R_cryst_,^(a)^ %	18.69
R_free_,^(b)^ %	22.53
RMS	
Bonds, Å	0.01
Angles, °	1.66
**Ramachandran Plot**	
Most favored, %	95.28
Additionally allowed, %	4.72
Disallowed, %	0
**PDB code**	6S0Y

(a) *R_cryst_ = Σ(|F_obs_|–|F_calc_|)/Σ|F_obs_|, F_obs_ and F_calc_ are observed and calculated structure factor amplitudes*.

(b) *R_free_ as for R_cryst_ using a random subset of the data excluded from the refinement*.

(c) *Data in brackets are for the highest resolution shell*.

### Docking

All water molecules, the ligand, and chain B of the nanobody crystal structure were manually deleted from the pdb-text file. The emptied structure was subjected to a local minimization with the GROMOS96 (43B1 parameter set) implementation within Swiss-PdbViewer 4.1.0 (41), and polar hydrogens were added. The peptide-ligand VETPIRNEWG was 3D-drawn with Avogadro 1.2.0 (42) and minimized with the built-in united force field. The AutoDockTools 1.5.6 suite (43) was used for pdbqt-format conversions and grid-box determination. The grid-box size was x = 22, y = 30 and z = 20 centered at x = 1.9, y = 9.7 and z = 8.3. Docking was performed with Smina (44, 45) with exhaustiveness set at 128. Visualization was with PyMOL 2.3.0 (46).

### Isothermal Titration Calorimetry

M2e-VHH-23m was dialyzed overnight against PBS buffer and concentrated using Amicon Ultra 3 kDa cut off centrifugal filter devices. The M2e peptide was resuspended in PBS at a stock concentration of 3 mM, and diluted in PBS to 300 μM. Titrations comprised 26 × 1.5 μL injections of peptide (300 μM) into the protein (30 μM), with 90 s intervals. An initial injection of ligand (0.5 μL) was made and discarded during data analysis. The data were fitted to a single binding site model using the Microcal LLC ITC_200_ Origin software provided by the manufacturer.

### Ala Scan Mutagenesis

HEK293T cells were transiently transfected with Flag-tagged M2 wild type (WT) and M2e Ala scan mutants. 24 h after infection the cells were detached, washed and blocked. Cells were stained with 20 μg/ml M2e VHH-23m or 20 μg/ml F-VHH-4 and subsequently fixed with 2% paraformaldehyde. After permeabilization (10 × permeabilization buffer diluted in double-distilled water; eBioscience), cells were stained with mouse anti-Histidine tag antibody (MCA1396, Abd Serotec) and rabbit anti-Flag tag antibody (F7425, Sigma-Aldrich). Binding of the primary antibodies was revealed with donkey anti-mouse IgG coupled to Alexa Fluor 647 (1/600; Invitrogen) and donkey anti-rabbit IgG coupled to Alexa Fluor 488 (1/600; Invitrogen). The median fluorescence intensity (MFI) of the cells was determined with an LSRII HTS flow cytometer (BD) and was calculated by subtracting the median fluorescence of binding of M2e-VHH-23m or F-VHH-4 to transfected cells from the median fluorescence of untransfected cells bound by M2e-VHH-23m or F-VHH-4.

### VHH Binding to Influenza A Virus Infected Cells

HEK293T cells were mock-infected or infected with A/Puerto Rico/8/1934 (H1N1), A/X47 (H3N2), A/Udorn/307/1972 (H3N2) or A/Swine/Ontario (H3N3) at a MOI of 1. Twenty-four hours after infection the cells were detached, washed and blocked. Cells were stained with 20 μg/ml M2e VHH-23m, 20 μg/ml F-VHH-4 or 10 μg/ml MAb148. To determine the affinity of M2e-VHH-23m on infected cells, 1/3 dilution series of M2e-VHH-23m or F-VHH4 were applied to A/Puerto Rico/8/1934 (H1N1) infected cells. Subsequently, the cells were fixed with 2% paraformaldehyde and stained with mouse anti-Histidine Tag antibody (MCA1396, Abd Serotec) and goat anti-A/Puerto Rico/8/1934 serum (Biodefense and Emerging Infections Resources Repository, NIAID, NIH, V314-511-157) followed by anti-mouse IgG Alexa 488 (Invitrogen) and anti-goat IgG Alexa 647 (Invitrogen). The median fluorescence intensity (MFI) was measured on the LSRII-tubes flow cytometer (BD) and was calculated by subtracting the median fluorescence of binding of M2e-VHH-23m or F-VHH-4 to infected cells from the median fluorescence of uninfected cells bound by M2e-VHH-23m or F-VHH-4.

### Plaque Reduction Assay

Different amounts of M2e-VHH-23m (2.5 μM, 1.25 μM or 0.625 μM), 0.333 μM MAb37 or sera of mice infected with A/Puerto Rico/8/1934 (H1N1) virus were incubated for 1 h at 4°C with 10–20 plaque forming units/well of A/Udorn/307/1972 (H3N2) or A/Puerto Rico/8/1934 (H1N1) virus. After incubation, the mixture was added to MDCK cells, seeded in a flat bottom 24-well plate. After 1 h, the cells were overlaid with an equal volume of 1.2% Avicel RC-591 (FMC Biopolymer) supplemented with 2 μg/ml of TPCK-treated trypsin (Sigma). Infection was allowed for 2 days at 37°C in 5% CO_2_. The overlay was subsequently removed and the cells were fixed with 4% paraformaldehyde. Viral plaques were stained with convalescent mouse anti- A/Puerto Rico/8/34 or A/Udorn/307/72 serum followed by horseradish peroxidase (HRP)-linked anti-mouse IgG (NXA931, GE Healthcare). Finally, after washing, the plaques were visualized with TrueBlue peroxidase substrate (KPL, Gaithersburg).

### VHH Binding to FcγRs Expressing Cells

Human Embryonic Kidney (HEK) 293T cells were transiently transfected with full length mouse FcγRI (MG50086-CF), FcγRIIb (MG50030-CY, SinoBiological Inc.), FcγRIII (MG50326, SinoBiological Inc.) or FcγRIV (MG50036-CF, SinoBiological Inc.) expression constructs along with the common γ-chain for the activating FcγRs (MG50935-CF) by polyethylenimine (PEI)-based transfection. A GFP-reporter plasmid was co-transfected. FcγRIV-VHH-7m and M2e-VHH-23m were directly labeled with the Alexa Fluor™ 647 antibody labeling kit (A20186, ThermoFisher scientific). Of the labeled VHHs, 0.2 μM or ¼ serial dilution series of the VHHs starting from 0.2 μM were added to the transfected cells or Mf4/4 cells (29). Fluorescence was measured on an LSRII flow cytometer (BD). The median fluorescence intensity (MFI) was calculated by subtracting the median fluorescence of binding of M2e-VHH-23m or FcγRIV-VHH-7m to transfected cells from the median fluorescence of untransfected cells bound by M2e-VHH-23m orFcγRIV-VHH-7m.

### Construction of Bispecific VHHs

To construct FcγRIV VHH-M2e VHH, FcγRIV VHH-F VHH, and FcγRIIIa VHH-M2e VHH bispecific VHHs, we made use of a GoldenBraid-based cloning strategy (47). The coding information of FcγRIV-VHH-7m or FcγRIIIa VHH [C28 sdAb, described by Behar et al. (48)] was amplified with the following forward (5′- GCG ATG CAG GGT CTC ACT TCA AGG CAG GTG CAG CTG CAG GAG TC-3′) and reverse primer (5′ GGC GAT GGT GGG TCT CAC TTC ATG AGG AGA CGG TGA CCT GGG-3′) that add specific overhangs for their identity as N-terminal VHH together with a *Bsa*I and *Sap*I restriction site. The C-terminal VHHs, M2e-VHH-23m and F-VHH-4 were amplified with a forward (5′-GCG CGA TGC AGG GTC TCA CTT CAC AGG TGC AGC TGC AGG AG TC-3′) and reverse primer (5′-GGG CGA TGG TGG GTC TCA CTT CAA GTC TAG TGA TGG TGA TGG TGG TGG CTG GAG ACG GTG ACC TG GG-3′) that add specific overhangs for their identity as C-terminal VHH together with a *Bsa*I and *Sap*I restriction site. The 15 amino acid long (Gly_4_Ser)_3_ linker was generated with the following forward (5′- GCG ATG CAG GGT CTC ACT TCA TCA GGC GGA GGC GGT AGT GGC GGA GGT GGA TCT GGA GGC GGC GGT AGT CA GT-3′) and reverse primer (5′- GGC GAT GGT GGG TCT CAC TTC ACT GAC TAC CGC CGC CTC CAG ATC CAC CTC CGC CAC TAC CGC CTC CGC CTG AT-3′) that also add a specific overhang together with a *Bsa*I and *Sap*I restriction site. The PCR amplified fragments were each assembled in a pUPD2 entry vector by a *Bsa*I restriction and ligation reaction. Once stored in the pUPD2 vector, the different parts were assembled together in the pKai61 expression vector using a T4 DNA ligase and a *Sap*I restriction enzyme which recognizes the *Sap*I restriction site which was introduced after ligation into the pUPD2 vector.

### *In vitro* FcγR Activation Assay

FcγR activation by MAbs 37 and 65 as well as by different M2e-specific nanobodies was determined using an *in vitro* FcγR activation assay (13, 30, 31). Cloning of FcγR-ζ constructs and the generation of FcγR-ζ BW5147 reporter cells were performed as reported previously (30, 31). Activation of stably transduced FcγR-ζ BW5147 reporter cells by immune complexes results in the production of mouse interleukin-2 (mIL-2), which was quantified by ELISA (30, 31).

HEK293T cells that were stably transfected with an M2 expression vector (28) were cultured in the presence of 10 μM amantadine. The cells were seeded 1 day before the co-culture experiment in 96-well flat-bottom plates, pre-coated with fibronectin purified from human plasma (4 μg/ml diluted in PBS). The next day, serial dilutions of the respective MAbs or VHH fusion constructs (concentrations as indicated ranging from 4 to 0.0625 μg/ml) were added to the HEK293T-M2 and incubated for 30 min at 37°C, followed by the addition of 1.5 × 10^5^ FcγR-ζ BW5147 reporter cells in a total volume of 200 μl RPMI1640 medium with 10% fetal calf serum per well. Target cells and reporter cells were incubated overnight at 37°C in a 5% CO_2_ atmosphere to allow mIL-2 production. Supernatants were analyzed by an anti-mIL-2 sandwich ELISA as described using the capture MAb JES6-1A12 and the biotinylated detection MAb JES6-5H4 (BD Pharmingen™, Belgium) (30, 31).

For infection with influenza A/Puerto Rico/8/1934 virus, Madin-Darby canine kidney (MDCK) cells were seeded in 96-well flat-bottom plates and infected with influenza PR8 virus (multiplicity of infection [MOI], 5). After 1 h of incubation at 37°C, unbound virus particles were removed by washing, and serial dilutions of the respective MAbs or VHHs (concentrations ranging from 4 to 0.0625 μg/ml) were added and incubated for 30 min at 37°C, followed by the addition of 1.5 × 10^5^ FcγR-ζ BW5147 reporter cells in a total volume of 200 μl RPMI1640 medium with 10% fetal calf serum per well. Target cells and reporter cells were incubated overnight at 37°C in a 5% CO_2_ atmosphere to allow mIL-2 production. Supernatants were analyzed by an anti-IL-2 sandwich ELISA as described (30, 31). If not indicated otherwise, experiments were performed in triplicates.

### Challenge Experiments in Mice

All experiments were approved by and performed according to the guidelines of the animal ethical committee of Ghent University (Ethical applications EC2017-66 and EC2018-12). Female BALB/c mice and male and female C57BL/6 mice were purchased from Charles River (France) and *Fc*γ*RIV*^−/−^ C57BL/6 mice (49) were bred in-house under specified-pathogen-free conditions. Mice were used at age 6–12 weeks and were SPF-housed with food and water *ad libitum*. Mice were anesthetized with isoflurane for treatment and infection. The mice were treated 4 h before and 24 h after influenza A virus challenge by intranasal administration of 50 μg of the bispecific VHHs in a volume of 50 μl PBS. Mice were challenged with 2xLD_50_ of A/X47 (H3N2) influenza virus. Body weight loss was monitored for 14 days. To determine the lung viral titer, complete lungs were harvested on day 6 after infection and homogenized in 1 ml of PBS with a sterile metal bead on the Mixer Mill MM 200 (Retsch). After clearance by centrifugation at 4°C, the lung homogenates were used for virus titration by plaque assay. The plaque assay was performed as described before, plaques were stained using convalescent mouse anti-X47 serum followed by horseradish peroxidase (HRP)-linked anti-mouse IgG (NXA931, GE Healthcare).

### Statistical Analysis

Statistical comparison of the differences in body weight loss was analyzed as repeated measurements data using the residual maximum likelihood (REML) as implemented in Genstat v19. Briefly, a linear mixed model (random terms underlined) of the form y = μ + experiment + treatment + time +treatment.time + mouse.time was fitted to the longitudinal data. The term mouse.time represents the residual error term with dependent errors because the repeated measurements are taken in the same individual, causing correlations among observations. Times of measurement were set as equally spaced, and the autoregressive model of order 1 was selected as the best correlation model based on the Aikake Information Coefficient. Significances of treatment effects across time (i.e., treatment.time) and of pairwise differences between treatment effects across time were assessed by an approximate *F*-test, of which the denominator degrees of freedom were calculated using algebraic derivatives as implemented in Genstat v19.

Survival analysis was performed on the right-censored survival data obtained for the five treatment groups. Groups were compared using the nonparametric log-rank test as implemented in Genstat v19. The two independent experiments were set as different groupings for a stratified test.

For the statistical analysis of the differences in lung viral titers a Hierarchical Generalized Linear Mixed Model (HGLMM; fixed model: poisson distribution, log link; random model: gamma distribution, log link) as implemented in Genstat v19 (see ref below), was fitted to the titer data. Treatment, having five levels, was set as fixed term, while replicate was set as random term. T statistics were used to assess the significance of treatment differences compared with the FcγRIV VHH-F VHH set as reference level (on the log-transformed scale). Estimated mean values and standard errors were obtained as predictions from the HGLMM, formed on the original scale of the response variable.

### Conservation of the M2 Ectodomain Sequences in Human H3N2 Influenza A Viruses

All complete M2 protein sequences of human H2N2 viruses, human H3N2 viruses and human H1N1 influenza A viruses circulating between 1933 and 2008 were extracted from the Influenza Research Database (http://www.fludb.org/) on 3th May 2019.

## Results

### Isolation of M2e-Specific VHHs

**Figure 1:**
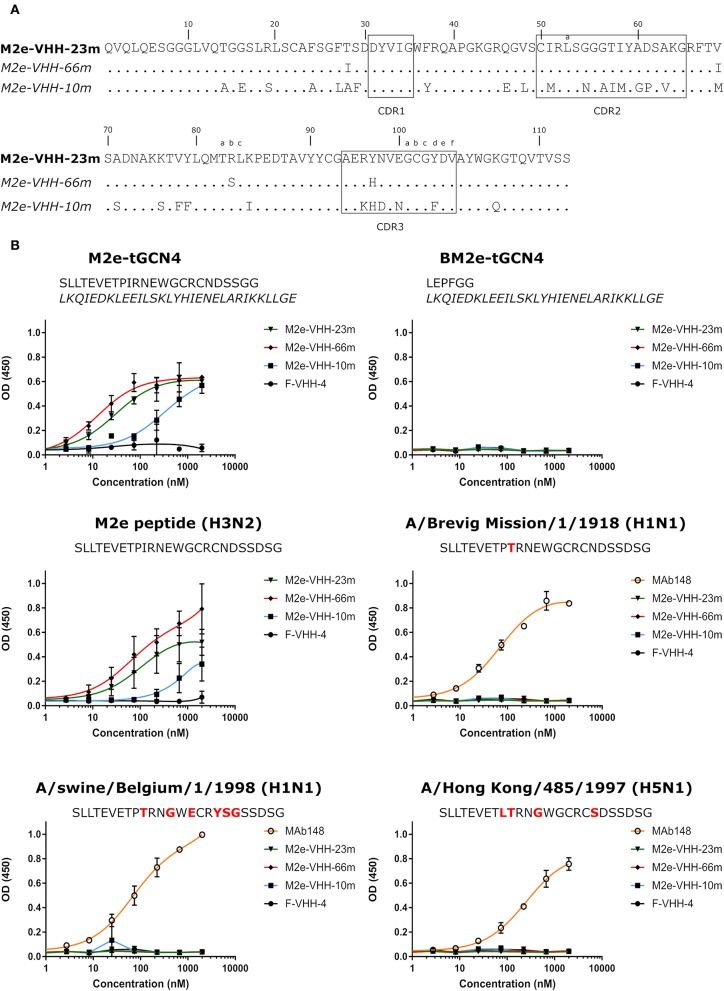
M2e-specific VHHs and their binding specificities. **(A)** Predicted amino acid residue sequences of M2e-VHH-23m and M2e-VHH-66m (isolated after panning on M2e-tGCN4 and human H3N2 M2e peptide) and M2e-VHH-10m (isolated after panning on HEK cells stably expressing M2). Above the sequences the Kabat numbering is indicated. The complementarity determining regions (CDR) are boxed. **(B)** M2e peptide ELISAs. Wells of microtiter plates were coated with 100 ng M2e-tGCN4, BM2e-tGCN4, or high-performance liquid chromatography (HPLC)-purified M2e peptide. The amino acid residue sequences of the coated proteins or peptides are depicted above the graphs. Amino acid residues that deviate from the consensus human H3N2 M2e sequence are highlighted in red. Dilution series of the indicated VHHs and MAb148 (a mouse monoclonal IgG1 that recognizes the M2e N-terminus) were added to the coated plates. Binding was detected with a mouse anti-His tag MAb, followed by a secondary sheep anti-mouse IgG Ab conjugated to horseradish peroxidase (HRP). Data points represent averages of triplicates and error bars represent standard deviations.

To generate M2e-specific VHHs a llama was immunized repeatedly with M2e-tGCN4 protein, a soluble recombinant immunogen that mimics the natural tetrameric M2e conformation, that was previously shown to induce protective M2e-specific IgG antibodies in mice (28, 50). Five days after the last immunization, peripheral blood lymphocytes were isolated from the llama and an immune VHH phagemid library of about 10^8^ clones was generated. M2e-specific VHHs were enriched from this library by sequential panning on immobilized M2e-tGCN4 (round 1) and M2e peptide (SLLTEVETPIRNEWGCRCNDSSD, corresponding to M2e of human H3N2 viruses) (round 2). As a second strategy, M2e-specific VHHs were enriched by panning on HEK293T cells that stably express M2. Individual phagemid clones enriched after both panning strategies were randomly selected and tested for binding to M2e-tGCN4 and M2e peptide in ELISA. Sequence analysis of the VHH encoding clones which tested positive for binding to either M2e-tGCN4 or M2e peptide revealed a low sequence diversity. Two clones (M2e-VHH-23m and M2e-VHH-66m) isolated with the first, and one clone (M2e-VHH-10m) isolated with the second panning strategy, were selected for further characterization. These VHHs had a cysteine residue at position 50 in the CDR2 and position 100b in the CDR3 (Kabat numbering), allowing the formation of an additional stabilizing disulfide bound, and were devoid of N-glycosylation sequons (Figure 1A) (51). The M2e-specific VHHs and an irrelevant control F-VHH-4 directed against the F protein of human respiratory syncytial virus, were subsequently expressed in *Pichia pastoris* in a secreted format (52). After purification from the yeast medium, the epitope specificity was assessed by ELISA. M2e-VHH-23m and M2e-VHH-66m bound to M2e-tGCN4 with a relatively high affinity, whereas M2e-VHH-10m displayed weaker binding (Figure 1B). None of the VHHs bound to purified recombinant influenza B M2 ectodomain fused to tGCN4, suggesting that the VHHs bind to M2e. The three selected VHHs bound to immobilized M2e-peptide in ELISA. Amino-acid residues 10 to 23 of M2(e) display some sequence diversity (50). We therefore tested binding of the VHHs to peptide variants that correspond to M2e from A/Brevig Mission/1918 (H1N1), A/Hong Kong/485/1997 (H5N1) and A/swine/Belgium/1/1998 (H1N1) (Figure 1B). None of the purified VHHs bound to these M2e peptides, whereas a control mouse monoclonal antibody (MAb148) that is specific for the extremely conserved amino-terminus of M2 (SLLTEVET) did bind (Figure 1B). This indicates that the isolated VHHs can bind to M2 expressed by human H2N2, the majority of human H3N2 strains and the majority of human H1N1 viruses circulating between 1933 and 2008 but are unlikely to recognize M2 of currently circulating human H1N1 viruses or most avian and swine influenza viruses. Remarkably, the isolated VHHs also failed to bind the H1N1 A/Brevig Mission/1/1918 M2e, which suggests that Ile11 in M2e contributes substantially to binding. We selected M2e-VHH-23m to target M2e in the subsequent experiments.

### M2e-Specific VHH Clamps M2e Peptide Between Its CDR2 and CDR3

**Figure 2:**
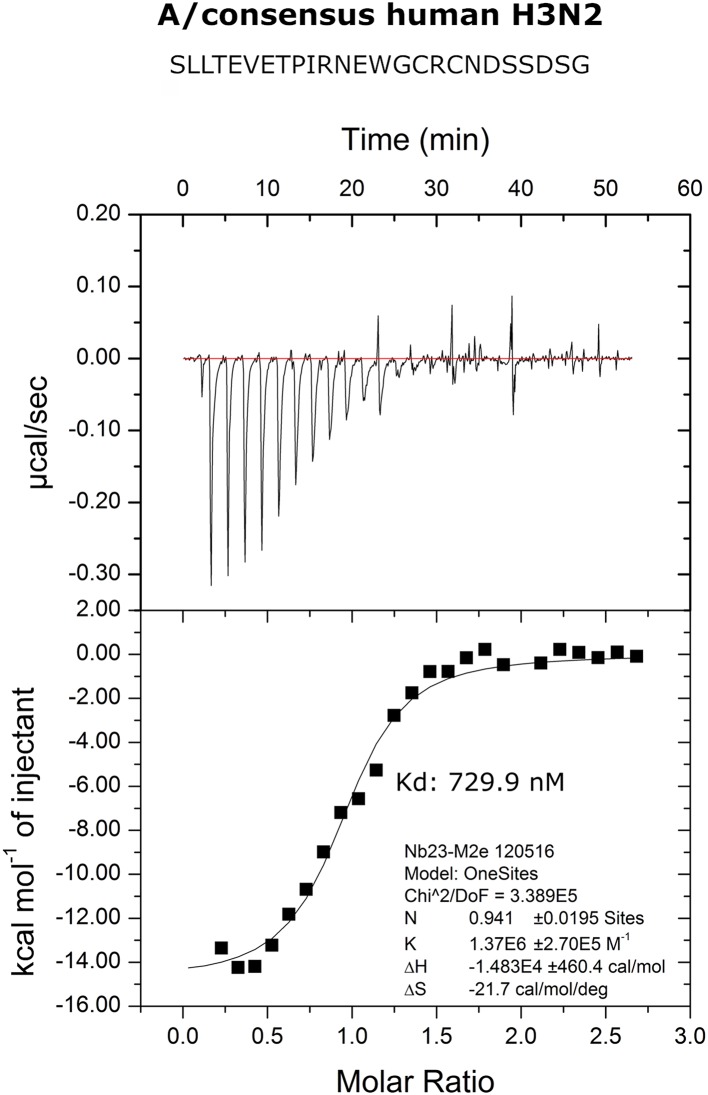
Thermodynamic characterization of the M2e:M2e-VHH-23m interaction. Isothermal titration calorimetry of the Me peptide into M2e-VHH-23m.

We performed isothermal titration calorimetry (ITC) experiments to determine the binding affinity of M2e-VHH-23m to the human H3N2 M2e peptide. The data revealed a 1:1 biomolecular association between the VHH and the M2e peptide with a K_d_ value of 730 nM (Figure 2). The affinity of the single domain M2e-VHH-23m is thus over a 1,000-fold lower than the previously reported affinities for the M2e-specific mouse monoclonal antibodies MAb65 and MAb37 (13).

**Figure 3:**
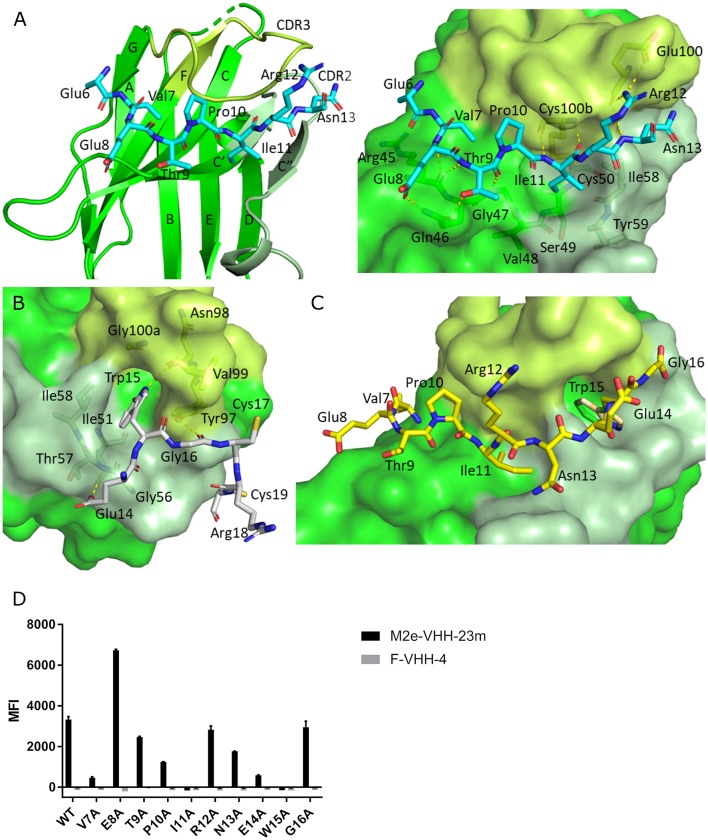
Molecular details of the M2e-VHH-23m: M2e interaction**. (A)** Crystal structure of M2e-VHH-23m in complex with the M2e peptide, showing the linear binding epitope of M2e residues 6–13 binding to a shallow groove on the surface of M2e-VHH-23m (Interface 1). Left: The M2e peptide is shown in cyan stick representation, the M2e-VHH-23m in green cartoon representation. CDR2 and CDR3 are colored pale and lime green, respectively. Right: Details of the interactions between M2e residues 6–13 and the residues making up interface 1 on M2e-VHH-23m. **(B)** Details of the interactions between M2e residues 14–19 and the residues making up interface 2 on M2e-VHH-23m. Coloring of M2e-VHH-23m as in A, M2e shown in gray stick representation. **(C)** Docking of the M2e peptide on the crystal structure of M2e-VHH-23m confirms the ligand swapping hypothesis, showing that M2e binds an extended grove on M2e-VHH-23m comprising both Interface 1 and Interface 2. Coloring of M2e-VHH-23m as in A, M2e shown in yellow stick representation. **(D)** Binding of M2e-VHH-23m to M2e Ala scan mutants. HEK293T cells were transfected with Flag-tagged M2 wild type (WT) and M2e Ala scan mutant expression constructs and subsequently incubated with 20 μg/ml of M2e-VHH-23m or F-VHH-4. After fixation with 2% paraformaldehyde and permeabilization, binding was detected with a mouse anti-Histidine tag antibody and rabbit anti-Fag tag antibody followed by an anti-mouse IgG Alexa 647 and anti-rabbit IgG Alexa 488, respectively. The median fluorescence intensity (MFI) was calculated by subtracting the median fluorescence of binding of M2e-VHH-23m or F-VHH-4 to transfected cells from the median fluorescence of non-transfected cells bound by M2e-VHH-23m or F-VHH-4.

To precisely resolve the epitope of M2e-VHH-23m, we determined the crystal structure of this VHH in complex with human H3N2 M2e peptide to 1.81 Å resolution (Table 1, PDB code 6S0Y). M2e-VHH-23m binds the M2e N-terminus as a linear epitope (Figure 3A, left panel). In the crystal structure, one M2e peptide interacts with three adjacent M2e-VHH-23m molecules at three respective interfaces (Figure S1). M2e residues E6 to N13 bind in a shallow groove formed by CDR3, CDR2 and the main body of the VHH formed by β-strands C, C', C”, and F (Interface 1; Figure 3A). As expected, based on the VHH sequence data, a stabilizing disulfide bridge is observed between the CDR3 and CDR2 (Figure 3A). Interface 1 includes hydrogen bonds between the M2e-VHH-23m residues Arg45-Gln46-Gly47 and M2e residues Glu8 and Thr9, M2e-VHH-23m residue Cys100b and M2e residue Ile11, and M2e-VHH-23m residue Glu100 and M2e residues Arg12 and Asn13. In addition, three hydrophobic contacts are formed: (1) M2e residue Val7 binds a shallow pocket lined by residue Phe37, Val100f and Trp103, (2) Pro10 stacks against Tyr100d, and (3) the side chain of M2e residue IIe11 is inserted in a pocket formed by the side chain of IIe58 of the VHH CDR2, and the backbones of Tyr59 (CDR2), Gly47, Val48 and Ser49 (CDR3). De bottom of the latter pocket is formed by the stabilizing disulfide bridge between Cys50 in CDR2 and Cys100b in CDR3 (Figure 3A). In interface 2, M2e residues 14 to 19 lie on a surface formed by CDR2 and CDR3. The highly conserved M2e Trp15 side chain is inserted in a deep pocket formed by residues from the 3 CDRs: the side chains of Ile58 and Tyr97, and backbones of Ile51, Gly56, Thr57, Asn98, Val99, and Gly100a. In addition, the imidazole NH of Trp15 is hydrogen bonded to the backbone of Asn98 (Interface 2, Figure 3B).

In the crystals, the M2e peptide contacts three adjacent molecules in an interaction reminiscent of domain swapping (53). Closer inspection indeed suggests that ligand swapping occurs between the M2e peptides bound to symmetry related molecules in the crystal (Figure S2). Docking experiments using the VETPIRNEWG M2e peptide show that the Interface 1 and 2 regions of the M2e peptide (residues 6–12 and 14–19, respectively) can bind a single M2e-VHH-23m molecule (Figure 3C). This is also supported by ITC experiments indicating that in solution M2e-VHH-23m and M2e interact in a 1:1 stoichiometry (Figure 2). To validate the relative importance of the two binding interfaces in this interaction, we additionally tested the binding of M2e-VHH-23m to HEK293T cells expressing M2 protein or M2e Ala-mutants by flow cytometry (Figure 3D). M2e-VHH-23m did not bind to cell that were transfected with an M2Ile11Ala or M2Trp15Ala expression construct, confirming the involvement of interface 1 and 2 in the interaction with M2e. These findings are also in line with the observation that M2e-VHH-23m fails to bind the H1N1 A/Brevig Mission/1/1918 M2e peptide, which carries a serine instead of an isoleucine residue at position 11 (Figure 1B). Reduced binding to cells expressing M2Val7Ala also concurs with the co-crystal structure data which show an interaction of M2eVal7 with a shallow hydrophobic pocket on the surface of M1e- VHH-23m (Figure 3A).

### M2e-VHH-23m Binds to Infected Cells and Does Not Neutralize Influenza A Virus

**Figure 4:**
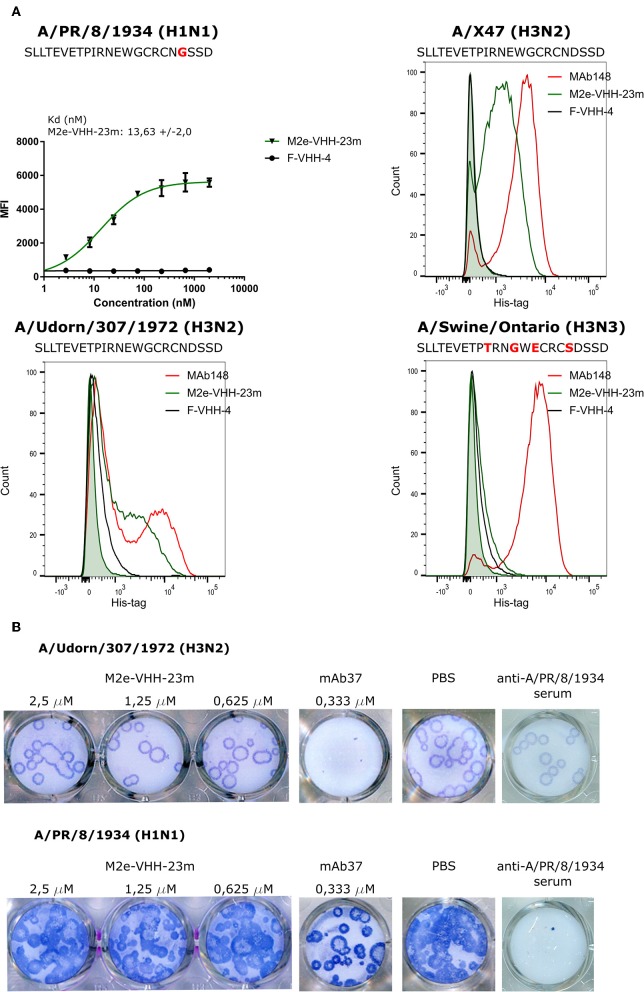
M2e-VHH-23m bind infected cells and lacks *in vitro* antiviral activity. **(A)** HEK293T cells were infected with A/Puerto Rico/8/1934 (H1N1), A/X47 (H3N2), A/Udorn/307/1972 (H3N2) or A/Swine/Ontario (H3N3) virus. The M2e sequences of the different viruses are shown above each graph, residues that differ from the consensus human H3N2 M2e sequence are colored red. Twenty-four hours after infection, the cells were stained with 20 μg/ml M2e VHH-23m, 20 μg/ml F-VHH-4 or 10 μg/ml MAb148. The A/Puerto Rico/8/1934 (H1N1) infected cells were stained with 1/3 dilution series of M2e-VHH-23m or F-VHH-4. After fixation with 2% paraformaldehyde, infected cells were stained with goat anti-A/Puerto Rico/8/1934 serum followed by anti-goat IgG Alexa 647 and bound VHHs were detected with a mouse anti-Histidine tag antibody followed by anti-mouse IgG Alexa 488. The mean fluorescence intensity (MFI) was calculated by subtracting the median fluorescence of binding of M2e-VHH-23m or F-VHH-4 to infected cells from the median fluorescence of uninfected cells bound by M2e-VHH-23m or F-VHH-4. Data points represent averages of triplicates and error bars represent standard deviations. Results of a representative of 3 repeat experiments is depicted for the binding to the A/Puerto Rico/8/1934 (H1N1) infected cells, the dissociation constant (Kd), is the average of three independent experiments together with the standard deviation. The green shading in the histograms corresponds to the binding of M2e-VHH-23m to mock-infected HEK cells. **(B)** M2e-VHH-23m (2.5, 1.25, or 0.625 μM), 0.333 μM MAb37 or sera of mice infected with A/Puerto Rico/8/1934 (H1N1) virus were incubated with 10 to 20 plaque forming units/well of A/Udorn/307/1972 (H3N2) or A/Puerto Rico/8/1934 (H1N1) virus and then added to MDCK cells. After 1 h, the cells were overlaid with Avicel supplemented with TPCK-treated trypsin. The overlay was removed after 2 days, cells were fixed with paraformaldehyde and viral plaques were stained with convalescent mouse anti- A/Puerto Rico/8/1934 or A/Udorn/307/1972 serum followed by HRP-linked anti-mouse IgG (NXA931, GE Healthcare) and TrueBleu substrate.

Next we used flow cytometry to determine if M2e-VHH-m23 could bind to M2e in its natural context, on the surface of influenza A virus infected cells. HEK293T cells were infected with A/Puerto Rico/8/1934 (H1N1) (M2e: SLLTEVETPIRNEWGCRCNGSSD), A/X47 (H3N2) (M2e: SLLTEVETPIRNEWGCRCNDSSD) or A/Udorn/307/1972 (H3N2) (M2e: SLLTEVETPIRNEWGCRCNDSSD) and were subsequently immuno-stained with MAb148, M2e-VHH-23m or F-VHH-4. M2e-VHH-23m could bind to the surface of cells infected with either of the three viruses with a K_d_ of 13.63 nM for influenza A/Puerto Rico/8/1934 (H1N1) infected cells (Figure 4A). In contrast, and as expected, M2e-VHH-23m failed to bind to cells infected with A/Swine/Ontario/42729A/2001 (H3N3) (M2e: SLLTEVETPTRNGWECRCSDSSD) virus. These data confirm that M2e-VHH-23m can bind to M2e sequences that are similar to the H3N2 M2e consensus sequence, recognizes the central part of such M2e sequences and that at least the aspartic acid to glycine substitution at position 21 in A/Puerto Rico/8/1934 M2 is not essential for binding.

It is known that some anti-M2e antibodies can restrict the *in vitro* replication of certain influenza A virus strains such as A/Udorn/307/1972 and A/Hong Kong/8/1968 but not other viruses such as A/Puerto Rico/8/1934 and A/WSN/1933 *in vitro* (54, 55). To address this issue, M2e-VHH-23m or MAb37, a M2e-specific IgG1 antibody with *in vitro* neutralizing activity against A/Udorn/307/1972, were mixed with 10–20 plaque forming units (pfu) of A/Puerto Rico/8/1934 or A/Udorn/307/1972 before infection of MDCK cells (56). As expected, both the plaque size and number of A/Udorn/307/1972, but not of A/Puerto Rico/8/1934 were reduced in the presence of MAb37 (Figure 4B). However, unlike MAb37, and even at a concentration of 2.5 μM (30 μg/ml), M2e-VHH-23m did not affect the number and size of A/Udorn/307/1972 plaques (Figure 4B).

### Isolation of FcγRIV-Specific VHHs

Our next aim was to arm M2e-VHH-23m with a second VHH that targets one of the activating FcγRs such as FcγRIV. We focused on this activating FcγR because of its restricted expression on macrophages, monocytes, neutrophils and dendritic cells but no other myeloid cell population, and because of its very high affinity for IgG2a monoclonal antibodies, which contribute the most protection by non-neutralizing influenza antibodies in the mouse model (13, 20).

**Figure 5:**
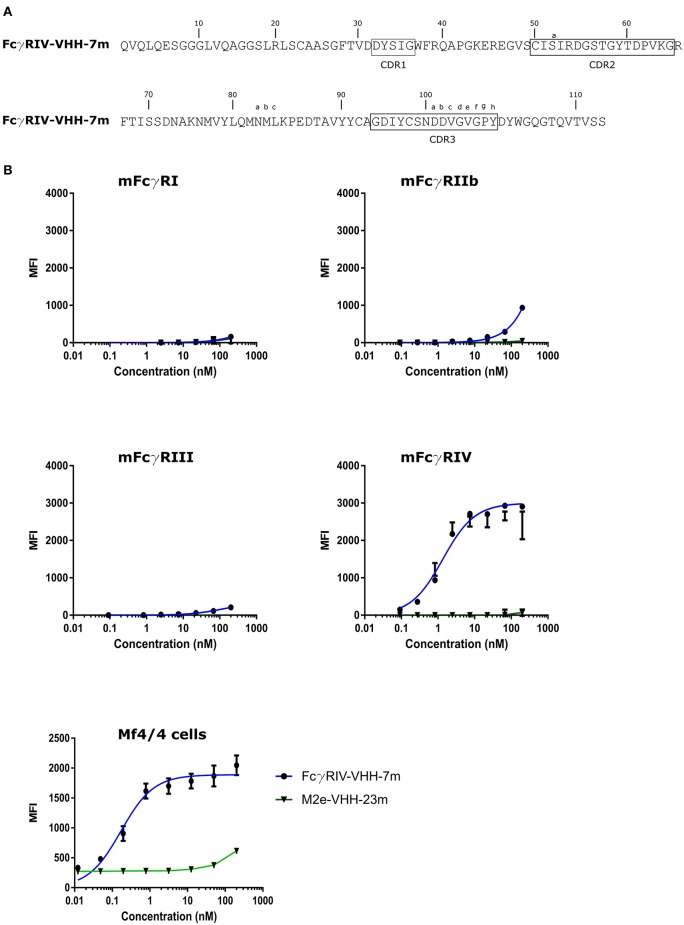
Characterization of the isolated FcγRIV-specific VHH. **(A)** The predicted amino acid residue sequence of FcγRIV-VHH-7m, isolated after panning on recombinant mouse FcγRIV protein of a VHH library derived from a llama immunized with immature mouse dendritic cells. Above the sequences the Kabat numbering is indicated. CDR1, −2, and −3 are boxed. **(B)** Flow cytometric analysis showing binding of FcgRIV-VHH-7m and M2e-VHH-23m to HEK 293T cells transiently transfected with expression vectors coding for mouse FcγRI, FγRIIb, FcγRIII, and FcγRIV along with the common γ-chain for the activating FcγRs and a GFP expression plasmid. The lower graph depicts binding to Mf4/4 cells. The cells were incubated with fourfold serial dilution of Alexa Fluor™ 647 labeled FcγRIV-VHH-7m or M2e-VHH-23m, starting at a concentration of 0.2 μM, binding of the VHHs to the GFP positive cells was analyzed. Data points represent averages of triplicates and error bars standard deviations.

To isolate FcγRIV-specific VHHs a phage library obtained from a llama that had been immunized with immature mouse dendritic cells (described by Deschacht *et al*.) was enriched for candidate FcγRIV-specific phagemid clones by three panning rounds using immobilized recombinant mouse FcγRIV extracellular domain protein produced in HEK293T cells (32). Of these candidates, FcγRIV-VHH-7m was selected after a subsequent ELISA screen using the FcγRIV antigen (Figure 5A). The binding specificity of purified FcγRIV-VHH-7m was analyzed by flow cytometry using HEK-293 cells that were transiently transfected with GFP and expression vectors coding for mouse FcγRI, FcγRIII or FcγRIV together with a plasmid coding for the common γ-chain, or with an expression vector coding for the inhibitory FcγRIIb. Concentration-dependent binding to cells transfected with the mouse FcγRIV plus the common γ chain was clearly detected (Figure 5B). Only at the highest concentration tested, FcγRIV-VHH-7m weakly bound to HEK293T cells that expressed mouse FcγRI, FcγRIIb or FcγRIII (Figure 5B). Expression of the FcγRs on the surface of the HEK293T cells was verified by staining with an antibody directed against the tag attached to the FcγRs (Figure S4). Clear expression of the mouse FcγRI, FcγRIII and FcγRIV was detected. Expression of the mouse FcγRIIb was less evident, therefore no firm conclusions about the binding of FcγRIV-VHH-7m to the mouse FcγRIIb could be deduced. To evaluate that FcγRIV-VHH-7m was able to bind to cells that express endogenous levels of the mouse FcγRIV, we tested its binding to Mf4/4 cells, a mouse macrophage like cell line (29). Unlike M2e-VHH-M2e, FcγRIV-VHH-7m bound Mf4/4 cells with a deduced K_d_ value comparable to the K_d_ value for binding to FcγRIV transfected cells (Figure 5B).

### Tail-to-Head Fused FcγRIV- and M2e-Specific VHHs Selectively and M2-Dependently Activate FcγRIV

**Figure 6:**
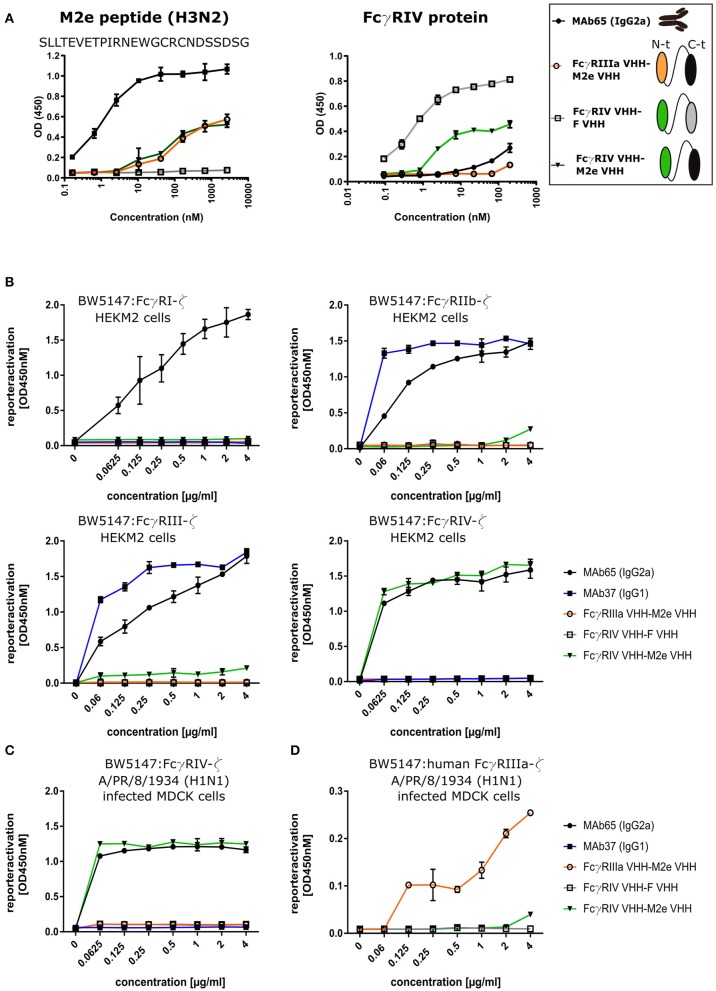
Bispecific fusion construct of anti-mouse FcγRIV VHH with M2e-VHH-23m selectively activates FcγRIV *in vitro*. **(A)** Schematic representation of the bispecific VHHs and ELISA on human H3N2 M2e peptide and recombinant FcγRIV protein is shown on the right. Wells of microtiter plates were coated with 100 ng peptide or protein. Dilution series of the bispecific VHH fusion constructs were added to the coated plates. Binding was detected with a mouse anti-His tag MAb, followed by a secondary sheep anti-mouse IgG Ab conjugated to HRP for the peptide ELISA. In the ELISA with coated recombinant FcγRIV protein, binding was detected with a HRP-conjugated rabbit anti-camelid VHH antibody. **(B)** Serial dilutions of the bispecific VHH fusion construct or monoclonal antibodies were added to HEK293T cells stably transfected with an influenza M2 expression plasmid. Thirty minutes later, FcγR-ζ BW5147 reporter cells were added to the HEK293T cells. After overnight incubation produced mIL-2 was measured in a sandwich-ELISA, which served as an indicator for the magnitude of FcγR activation. (C) MDCK cells were infected with A/Puerto Rico/8/1934 (H1N1) virus for 1 h. Unbound virus particles were washed away and serial dilutions of the bispecific VHHs or monoclonal antibodies were added and incubated for 30 min, followed by the addition of the FcγRIV-ζ BW5147 reporter cells **(C)** or human FcγRIIIa-ζ BW5147 reporter cells **(D)**. After overnight incubation supernatants were analyzed by an anti mIL-2 sandwich ELISA. Data points represent averages of triplicates and error bars represent standard deviations. The graphs are a representative of one out of three repeat experiments.

To evaluate the possibility to arm the M2e-specifc VHH with FcγRIV-dependent effector functions, we genetically fused M2e-VHH-23m carboxy-terminal of FcγRIV-VHH-7m by means of a flexible 15 amino acid long (Gly_4_Ser)_3_ linker. As controls M2e-VHH-23m was also fused to a VHH directed against human FcγRIIIa [described by Behar et al. (48)] and FcγRIV-VHH-7m was linked to F-VHH4. The resulting constructs were named FcγRIV VHH-M2e VHH, FcγRIIIa VHH-M2e VHH, and FcγRIV VHH-F VHH, respectively, and were produced in transformed *Pichia pastoris* shake flask cultures (Figure 6A). FcγRIV VHH-M2e VHH could bind to M2e peptide and coated soluble FcγRIV protein in ELISA (Figure 6A). Next, we evaluated the potency of the bispecific constructs to activate individual FcγRs *in vitro*, by making use of a co-culture of M2-expressing cells and a set of reporter cell transfectants, which produce interleukin 2 upon the activation of a specific FcγR (Figure 6B) (30). As controls for FcγR activation, we used mouse M2e-specific MAb 37 (IgG1) and MAb 65 (IgG2a). In the presence of M2-expressing HEK293T cells, MAb 65 dose-dependently activated all mouse FcγRs. This is expected as mouse IgG2a antibodies can bind and activate all mouse FcγRs (16). Only mouse FcγRIIb and -RIII were activated by MAb37, again in line with the known binding specificity of mouse IgG1 antibodies for mouse FcγRs (15). In the presence of M2-expressing cells FcγRIV VHH-M2eVHH potently activated mouse FcγRIV but not FcγRI, -III, and -IIb. None of the control VHH fusion constructs activated any of the FcγRs, indicating that specificity for both M2e and FcγRIV of the VHH fusion constructs was required. Moreover, activation of the mouse FcγRIV reporter cells by FcγRIV VHH-M2eVHH was only observed in the presence of M2 expressing target cells (Figure S5). Potent and dose-dependent activation of mouse FcγRIV by the FcγRIV VHH-M2e VHH fusion was also observed when A/Puerto Rico/8/1934 infected MDCK cells were used as target cells (Figure 6C). Finally, FcγRIIIa VHH-M2e VHH, but not FcγRIV VHH-M2eVHH, activated the human FcγRIIIa, the ortholog of mouse FcγRIV, in the presence of A/Puerto Rico/8/1934 infected targeted cells (Figure 6D). We conclude that the tail-to-head fusion of the FcγRIV- with the M2e-specific VHHs allows selective, M2-dependent *in vitro* activation of the mouse FcγRIV.

### Intranasal Administration of Bispecific FcγRIV VHH-M2e VHH Protects Mice From a Potentially Lethal Influenza A Virus Challenge

**Figure 7:**
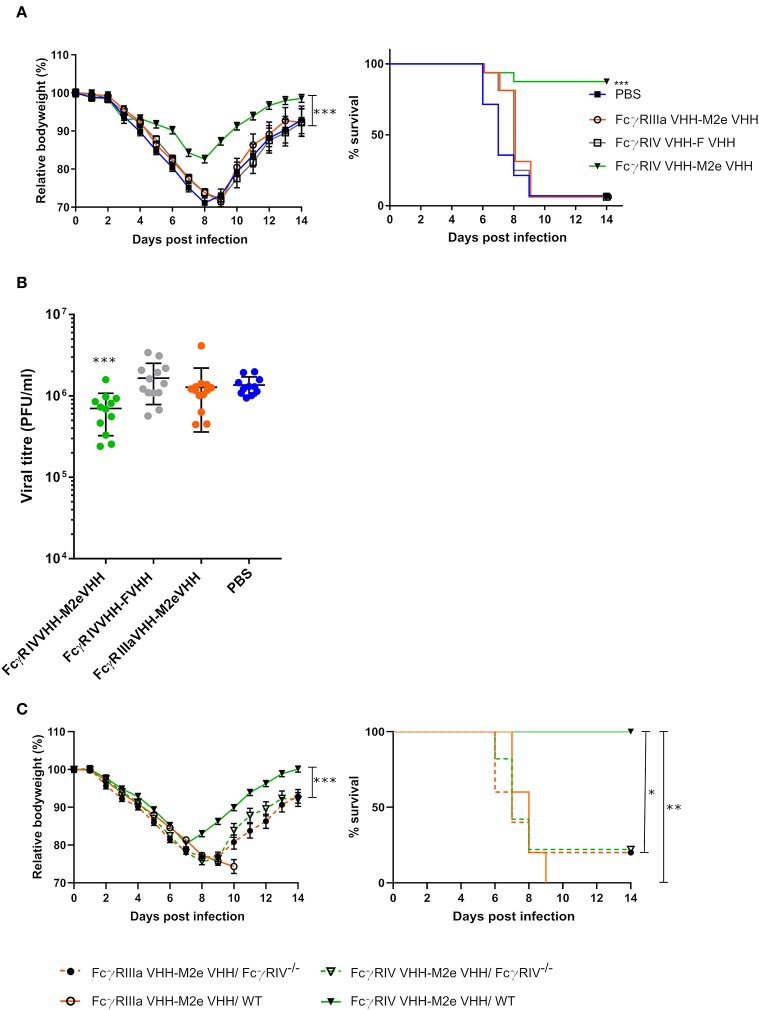
Bispecific fusion construct of a FcγRIV- and M2e-specific VHH protects mice against a potentially lethal influenza A virus infection. **(A)** Groups of 16 (bispecific VHHs) or 14 (PBS) BALB/c mice were intranasally treated with 50 μg of the bispecific VHHs or PBS 4 h before and 24 h after challenge with 2xLD_50_ of A/X47 (H3N2) influenza virus. Body weight change (left) and survival (right) were monitored for 14 days. The mean relative changes in body weight together with their standard errors, are represented. The difference in body weight loss between FcγRIV VHH-M2e VHH and the negative-control groups was statistically significant (***P < 0.001, REML variance components analysis). The survival rate of the group receiving FcγRIV VHH-M2e VHH was significantly different from FcγRIV VHH-F VHH control treatment group (***p < 0.001, Log-rank test). **(B)** To determine the effect on the viral load in the lungs, groups of 13 (bispecific VHHs) or 11 (PBS) BALB/c mice received 50 μg of the bispecific VHHs or PBS 4 h before and 24 h after viral challenge with 2xLD_50_ of A/X47 (H3N2) influenza virus. On day 6 after infection, the lungs were harvested and the viral titer was determined by plaque assay. The viral titer of mice FcγRIV VHH-M2e VHH was significantly different compared to mice that received the FcγRIV VHH-F VHH control treatment (***P < 0.001, unpaired *t*-test). **(C)** Groups of 5 wild-type and *Fc*γ*RIV*^−/−^ C57BL/6 mice (male and female mice) were treated with 50 μg of the bispecific VHHs 4 h before and 24 h after viral challenge with 2xLD_50_ of A/X47 (H3N2) influenza virus and body weight (left) and survival (right) were monitored. In the left-hand graph, data points represent means and error bars represent the standard errors of the means. Body weight changes between FcγRIV VHH-M2e VHH treated wild-type and FcγRIV^−/−^ mice were significantly different (***P < 0.001, REML variance components analysis). The survival rate of wild-type mice that received FcγRIV VHH-M2e VHH was significantly different from wild-type mice treated with FcγRIIIa VHH-M2e VHH (**P < 0.01, REML variance components analysis) and FcγRIV^−/−^ mice treated with FcγRIV VHH-M2e VHH and FcγRIIIa VHH-M2e VHH (*P < 0.05, REML variance components analysis). Data in **(A,B)** are pooled from 2 independent experiments.

In a final set of experiments, we explored the protective potential of FcγRIV VHH-M2e VHH *in vivo*. SPF-housed female BALB/c mice were treated intranasally with 50 μg FcγRIV VHH-M2e VHH 4 h before and 24 h after challenge with 2xLD_50_ of A/X47 (H3N2) influenza virus. As controls, mice were treated with PBS, 50 μg of FcγRIIIa VHH-M2e VHH or FcγRIV VHH-F VHH. The mice that had been treated with FcγRIV VHH-M2e VHH were significantly better protected from body weight loss and lethality caused by the influenza virus infection compared, to mice that had been treated with the negative control VHH fusion constructs, which combine targeting of FcγRIIIa with M2e-specificity or targeting of RSV-F with FcγRIII-specificity (Figure 7A). In addition, the protection mediated by FcγRIV VHH-M2e VHH was associated with a modest but statistically significant reduction in lung viral titer (Figure 7B). To verify if the protection by FcγRIV VHH-M2e VHH was mediated by FcγRIV engagement, wild-type and *Fc*γ*RIV*^−/−^ C57BL/6 mice were intranasally treated with 50 μg FcγRIV VHH-M2e VHH or FcγRIIIa VHH-M2e VHH, as negative control, 4 h before and 24 h after challenge with 2xLD_50_ of A/X47 (H3N2) influenza virus. While treatment with FcγRIV VHH-M2e VHH could protect wild-type mice, *Fc*γ*RIV*^−/−^ mice and control-treated wild-type mice did not survive the virus challenge (Figure 7C). Moreover, body weight loss after challenge was statistically significantly reduced in FcγRIV VHH-M2e VHH treated wild-type mice, compared to *Fc*γ*RIV*^−/−^ mice (*p* < 0.001). Thus, selective targeting of mouse FcγRIV with the recombinant two-domain construct FcγRIV VHH-M2e VHH, can protect mice against a potentially lethal influenza A virus challenge.

## Discussion

Formatting of VHH fragments to develop biologicals to prevent or treat infectious diseases is a powerful and attractive approach to develop biologicals with enhanced specificity, activity, half-life and breadth of protection. Here, VHH formatting was used to test if arming of a virus surface antigen-specific VHH with an artificial and selective FcγR activation function can potentiate its activity to combat viral infections in the absence of direct virus neutralization. To explore this possibility, we focused on influenza A M2e as a viral target. This antigen is conserved among influenza A viruses and is therefore an attractive target to achieve broad protection. M2e-specific VHHs were obtained after immunization of a llama with M2e fused to a heterologous tetramerizing domain, to mimic the natural quaternary structure of M2, followed by panning on M2e peptide or cells that stably express M2. We isolated and characterized in more detail three M2e-specific VHHs. Although several M2e-specific conventional MAbs have been described in recent years, to our knowledge, these are the first reported single domain antibodies that specifically bind to M2e. Recently, a M2-specific VHH was isolated from a synthetic VHH library by Wei et al. (57), however their VHH failed to bind to M2e peptide.

We determined the crystal structure of M2e-VHH-23m in complex with a M2e peptide, that corresponds to the consensus sequence of human H1N1 viruses that circulated between 1933 and 2008, human H2N2 and H3N2 viruses. This structure revealed that the M2e antigen is bound by the VHH in an extended conformation that wraps around two main interfaces on the VHH. A first interface is a shallow groove formed by the CDR2 and CDR3 loops and the C, C', C”, and F β-strands (Figure 3A). This interface binds the M2e region spanning residues 6–12, where Val7, Pro10 and Ile 11 bind hydrophobic patches on the VHH. In addition to three main chain interactions, the side chains of M2eGlu8 and -Thr9 each go into an H-bond interaction with the VHH. A second binding interface is found on the “back” side of the VHH, interacting with residues 14–19 of M2e. This interface contains a well-defined hydrophobic pocket that captures the side chain of the highly conserved Trp15. Most avian and swine influenza A viruses, and, since the 2009 pandemic, also most circulating human H1N1 viruses, have a threonine instead of an isoleucine at position 11 of M2(e). Threonine is not hydrophobic and would be incompatible to the VHH's hydrophobic pocket. This likely explains why M2e-VHH-23m fails to bind to the tested avian and swine M2e peptide variants. In contrast to Ile11, M2e-Trp15, which occupies the second hydrophobic pocket of the VHH, is strictly conserved in all known influenza A viruses. M2e Ala-scan analysis revealed that next to Ile11 and Trp15 also substitution of Val7 or Glu14 considerably impairs binding of M2e-VHH-23m. M2eVal7 is strictly conserved in all influenza A viruses and M2eGlu14 is highly conserved in human influenza A viruses. Since M2eVal7, -Ile11, -Glu14, and -Trp15 are highly conserved in all H3N2 influenza A strains, M2e-VHH-23m might have the potential to recognize the vast majority of these strains. The M2e N-terminus (SLLTEVET) is highly conserved among all influenza A viruses and is recognized by some conventional monoclonal antibodies such as MAb 148 and TCN-032. Despite employing various panning strategies using avian and swine M2e variants, we could not yet isolate a VHH that specifically binds the strictly conserved region in the M2e N-terminus.

Earlier we reported the crystal structures of M2e in complex with the Fab fragment of two different mouse monoclonal antibodies (MAb 65 and MAb 148) that recognize partially overlapping epitopes of M2e (50, 58). In these two crystal structures, M2e adopted two different conformations. The epitopes of the MAb 65 and M2e-VHH-23m are highly similar: they both span the TEVETPIRNEW fragment and in both M2eIle11 and -Trp15 are important for binding. Nevertheless, the conformation of the TEVETPIRNEW part of M2e in complex with M2e-VHH-23m is very different from that in complex with the MAb 65 (Figure S3). This indicates that M2e indeed has little intrinsic structure and can adopt multiple conformations dependent on the bound antibody. This flexible nature of the M2e peptide could explain why it appears to be difficult to isolate a VHH that recognizes the N-terminal part of M2e. Moreover, due to the absence of the light chain, VHHs typically bind to concave epitopes rather than to convex or protruding peptide termini (59, 60). Nevertheless a few VHHs directed against linear peptides have been described (61, 62). De Genst et al. reported on a VHH that binds to the C-terminus of the intrinsically disordered a-synuclein protein with nanomolar range affinity (61). The crystal structure revealed that this VHH binds the four C-terminal amino acids of α-synuclein by a narrow pocket formed by its CDR2 and CDR3. This suggests that it may be possible to isolate VHHs that specifically bind the highly conserved N-terminus of M2.

In this study, we also report the isolation of a FcγRIV-specific VHH from a llama that had been immunized with immature mouse dendritic cells. This VHH bound to mouse FcγRIV and showed no cross reactivity with the other FcγRs. To our knowledge, this is the first report of an FcγRIV-specific VHH. A fibronectin scaffold protein that could specifically bind to FcγRIV with high affinity has been reported (63). In addition, this scaffold protein was able to delay tumor growth in a mouse model when linked to an anti-tumor antigen-specific single-chain antibody and mouse serum albumin (to extend the half-life of the fusion construct).

The bivalent VHH comprising the FcγRIV-specific VHH and the M2e-specific VHH, could activate the mouse FcγRIV in the presence of influenza A virus infected cells. Specifically targeting only FcγRIV might be an advantage over conventional antibodies since the activation of the inhibitory FcγRIIb receptor, which dampens the immune cell activation, is circumvented this way. In this context, other strategies such as site-directed mutagenesis, bispecific antibody formats and glycan engineering have been explored to try to increase Fc binding to activating receptors and decrease the interaction with the inhibitory receptor FcγRIIb (64–67). For example, afucosylated antibodies against RSV, Ebola virus and HIV with enhanced FcγR binding showed enhanced efficacy in rodent models (67–69).

When administered 4 h before and 24 h after infection, the bispecific FcγRIV VHH-M2e VHH construct protected wild-type but not *Fc*γ*RIV*^−/−^ mice against an otherwise lethal influenza A virus challenge. It is well documented that protection by M2e-specifc antibodies is FcγR mediated (6–9). We recently reported that wild type mice treated with an M2e-specific IgG2a showed significantly less body weight loss after infection compared with *Fc*γ*RIV*-/- mice, suggesting that FcγRIV could contribute to protection by M2e-specific IgG2a (13). Here we show that the selective activation of the mouse FcγRIV with a bispecific VHH fusion that also binds to M2e is sufficient to protect mice against a potentially lethal influenza A virus challenge. Antitumor activity has been reported for bispecific VHH fusion constructs that consist of a VHH directed against the human FcγRIIIa (the ortholog of the mouse FcγRIV), fused to either a VHH directed against carcinoembryonic antigen (CEA) or HER2 (70, 71). In the context of viral infections, it has been reported that selective engagement of human FcγRIIIa could mediate killing of HIV infected target cells by NK cells (72, 73). It remains to be investigated whether exchanging the mouse FcγRIV specific VHH by a human FcγRIIIa specific VHH in the bispecific fusion construct described here, would be able to protect against influenza A virus infection in the context of a human FcγR repertoire. Moreover, the M2e-specific VHH could be exchanged by a VHH direct against another (conserved) viral target antigen such as the hemagglutinin stalk domain, since FcγRs also seem to play an important role in the protection mediated by anti-stalk antibodies (74, 75).

In summary, we have demonstrated that an intranasally administered, bi-specific VHH fusion construct that selectively binds to and activates FcγRIV with one moiety and M2e as present on infected target cells can protect against influenza A virus challenge. However, the treated mice still showed substantial bodyweight loss following challenge whereas intranasal administration of a M2e-specific IgG2a monoclonal antibody largely controlled the morbidity following challenge of the mice (unpublished result). In future studies one could try to optimize the Fcγ Receptor engaging VHH-fusions by extending their lung retention, and further increasing the affinity for both the viral target and the specific FcγR. Finally, when humanized, formatted M2e-specific VHHs might provide a new treatment option in the battle against influenza A virus infections. This approach could also be of interest for other viral infections. Such VHHs might be especially well suited to prevent or treat respiratory infections because VHHs can be delivered directly to the site of infection as inhaled biotherapeutics by nebulization (27).

## Data Availability Statement

All datasets generated for this study are included in the article/Supplementary Material, and will also be made available by the authors, without undue reservation, to any qualified researcher.

## Ethics Statement

All mouse experiments complied with national (Belgian Law 14/08/1986 and 22/12/20333, Belgian Royal Decree 06/04/2010) and European legislation (EU Directives 2010/63/EU and 86/609EEG) on animal regulations. All experiments were approved by and performed according to the guidelines of the animal ethical committee of Ghent University (permit number LA1400091, ethical applications EC2017-66 and EC2018-12).

## Author Contributions

DD, BS, and XS planned the study. DD, KH, IV, WN, LV, MB, SC, and BS performed the research. DD, BS, IV, and XS wrote the manuscript with contributions from KH, WN, HR, and HH. All authors reviewed the manuscript before submission.

### Conflict of Interest

The authors declare that the research was conducted in the absence of any commercial or financial relationships that could be construed as a potential conflict of interest.
