# Supplementary material for: Selective Engagement of FcγRIV by a M2e-Specific Single Domain Antibody Construct Protects Against Influenza A Virus Infection
Source: Front Immunol. 2019 Dec 12;10:2920. doi: 10.3389/fimmu.2019.02920 (PMC6921966; doi:10.3389/fimmu.2019.02920)
Supplement: Supplementary file 1 [file Data_Sheet_1.PDF]

Supplementary material

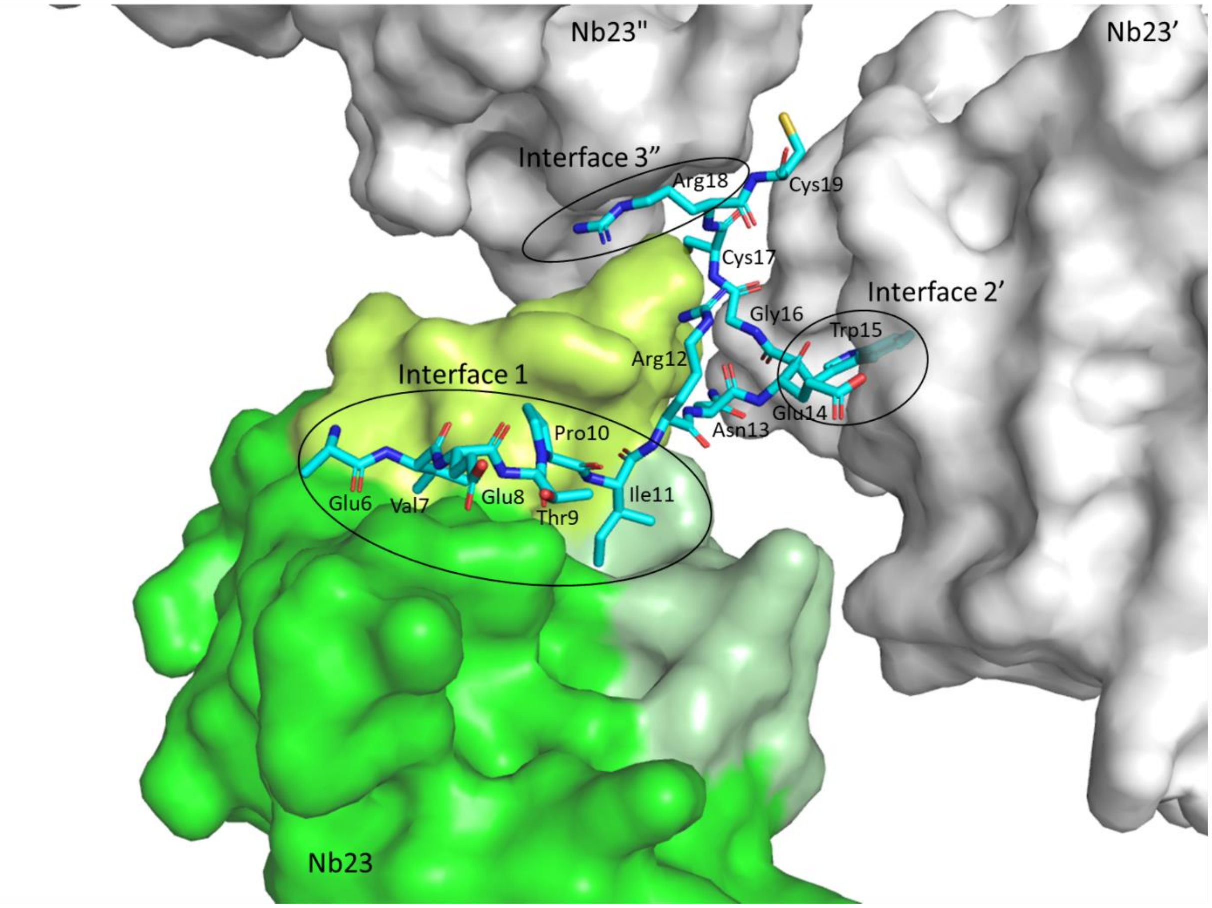

Figure S1. Crystal structure of M2e-VHH-23m in complex with the M2e peptide, showing that the peptide is bound to 3 M2e-VHH-23m molecules. M2e-VHH-23m is shown in surface representation. The canonical M2e-VHH-23m in the asymmetric unit (Nb23) is colored green, its CDR2 and CDR3 are shown in pale and lime green, respectively. Two symmetry related M2e-VHH-23m molecules, Nb23’ and Nb23”, are shown in light grey. The M2e peptide is shown in cyan stick representation.

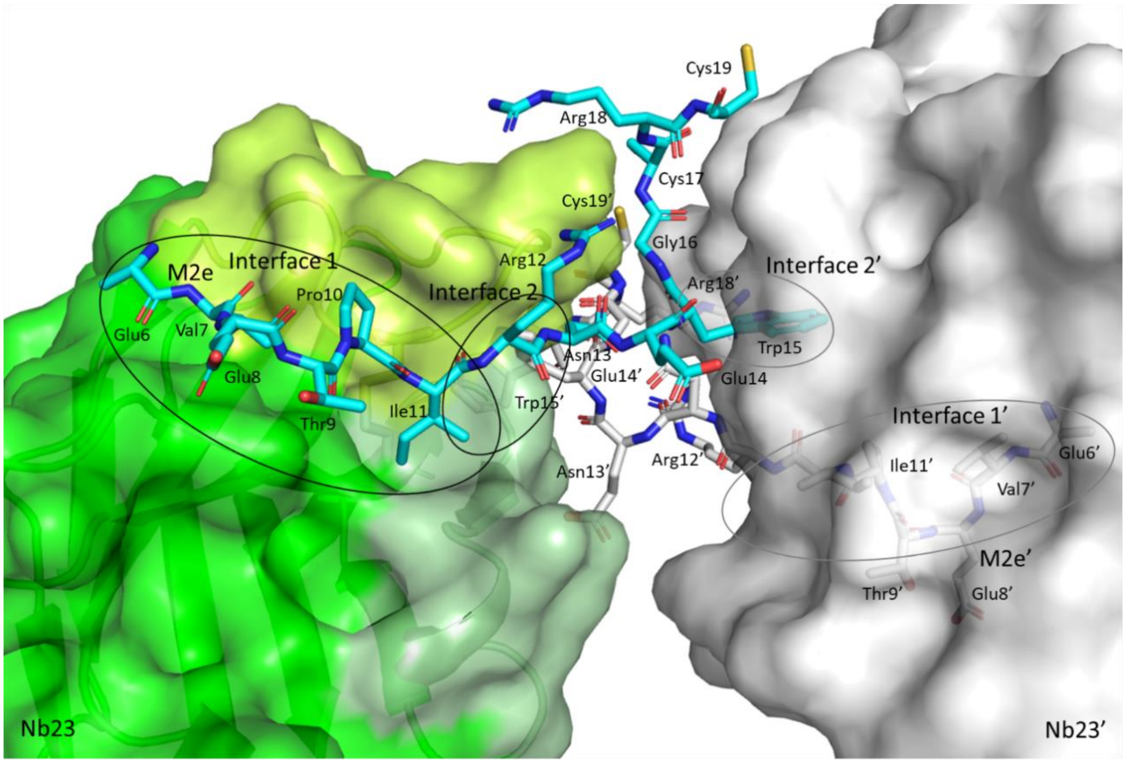

Figure S2. M2e ligand swapping. The crystal structure of M2e-VHH-23m in complex with the M2e peptide shows that ligand swapping occurs between two peptides bound to symmetry related M2e-VHH-223m molecules. M2e-VHH-23m is shown in surface representation. The canonical M2e-VHH-23m in the asymmetric unit (Nb23) is colored green, its CDR2 and CDR3 are shown in pale and lime green, respectively. The symmetry related M2e-VHH-23m Nb23 is shown in light grey. The M2e peptide bound to Nb23 is shown in cyan stick, the one to Nb23' in grey stick representation.

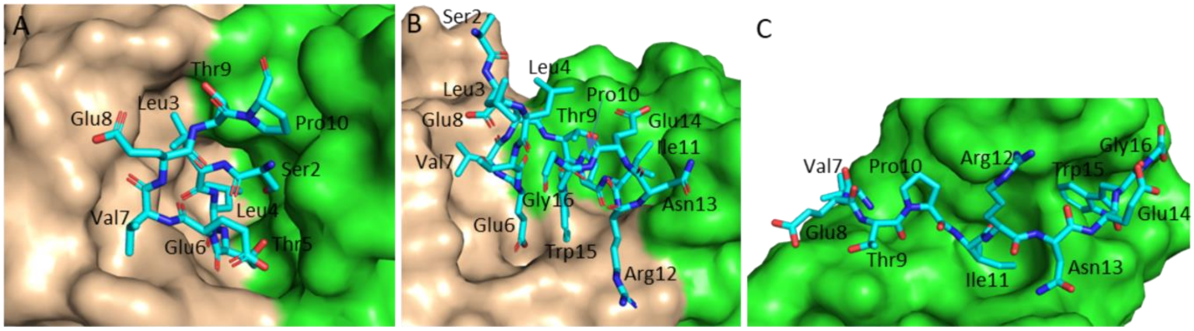

Figure S3. Comparison between conformation adopted by M2e when bound to MAb148 (A, PDB code 5DLM), MAb65 (B, PDB code 4N8C) and M2e-VHH-23m (C, docking model also shown in Fig. 3C). The antibodies are shown in surface representation, while the M2e peptide is shown as sticks. For the monoclonal antibodies, the light chain is shown in green, the heavy chain in wheat. The M2e-VHH-23m VHH is shown in green.

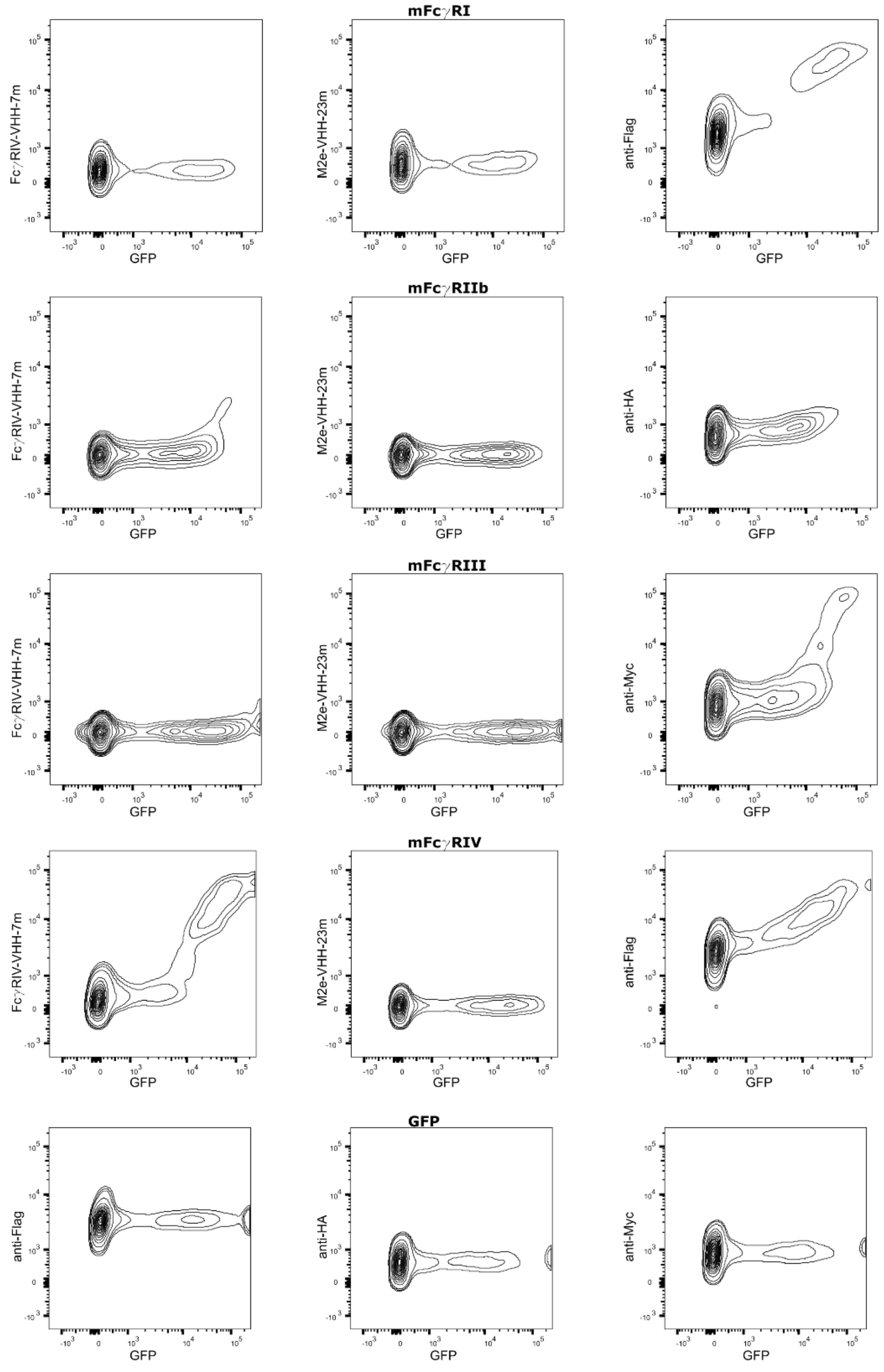

Figure S4. Fcγ receptor binding specificity of FcγRIV-specific VHH. HEK 293T cells were transiently transfected with GFP and an expression vector encoding for mouse FcγRI, FcγRIIb, FcγRIII and FcγRIV along with the common γ-chain for the activating FcγRs. Cells were stained with 0. 1 µM Alexa Fluor™647 labeled FcγRIV-VHH-7m or M2e-VHH-23m or an antibody directed against the tag attached to the FcγRs (FcγRI and FcγRIV: Flag-tag; FcγRIIb: HA-tag; FcγRIII: Myc-tag).

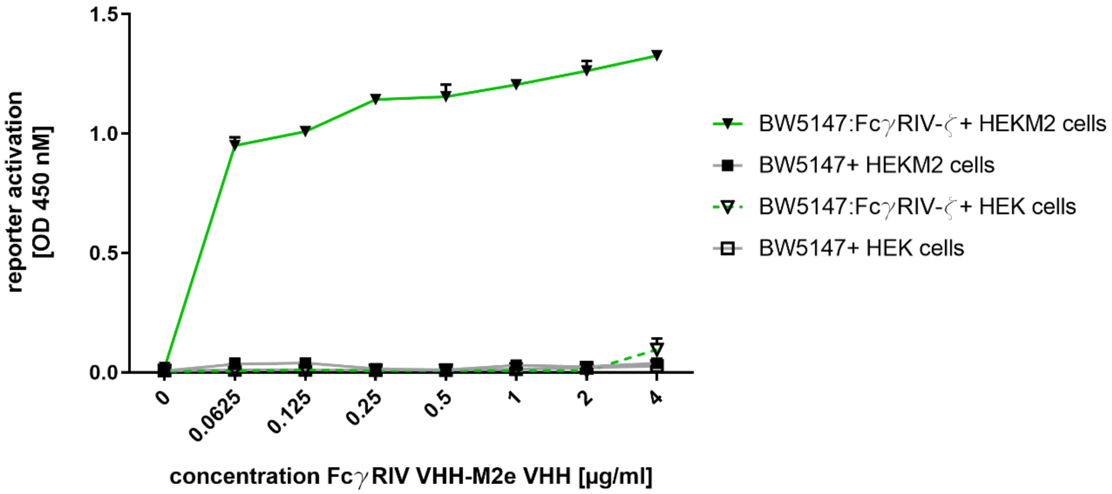

Figure S5. Bispecific fusion construct of anti-mouse FcγRIV VHH with M2e-VHH-23m only activates FcγRIV in vitro, in the presence of M2 expressing cells. Serial dilutions of the FcγRIV VHH-M2e VHH bispecific fusion construct were added to HEK293T cells or HEK293T cells stably transfected with an influenza M2 expression plasmid. Thirty minutes later, FcγRIV-ζ BW5147 reporter cells or parental BW5147 reporter cells (which do not express FcγRs) were added to the HEK293T cells. After overnight incubation produced mIL-2 was measured in a sandwich-ELISA, which served as an indicator for the magnitude of FcγR activation. Data points represent averages of triplicates and error bars represent standard deviations. The graph is a representative of one out of two repeat experiments.
